# Effective medium theory for bcc metals: electronically non-adiabatic H atom scattering in full dimensions

**DOI:** 10.1039/d2cp00087c

**Published:** 2022-04-04

**Authors:** Nils Hertl, Alexander Kandratsenka, Alec M. Wodtke

**Affiliations:** Max-Planck-Institut für Multidisziplinäre Naturwissenschaften Am Faßberg 11 Göttingen Germany nils.hertl@mpinat.mpg.de; Institut für Physikalische Chemie, Georg-August-Universität Tammannstraße 6 Göttingen Germany; International Center for Advanced Studies of Energy Conversion, Georg-August-Universität Tammannstraße 6 Göttingen Germany

## Abstract

We report a newly derived Effective Medium Theory (EMT) formalism for bcc metals and apply it for the construction of a full-dimensional PES for H atoms interacting with molybdenum (Mo) and tungsten (W). We construct PESs for the (111) and (110) facets of both metals. The EMT-PESs have the advantage that they automatically provide the background electron density on the fly which makes incorporation of ehp excitation within the framework of electronic friction straightforward. Using molecular dynamics with electronic friction (MDEF) with these new PESs, we simulated 2.76 eV H atoms scattering and adsorption. The large energy losses at a surface temperature of 300 K is very similar those seen for H atom scattering from the late fcc metals and is dominated by ehp excitation. We see significant differences in the scattering from different surface facets of the same metal. For the (110) facet, we see strong evidence of sub-surface scattering, which should be observable in experiment and we predict the best conditions for observing this novel type of scattering process. At low temperatures the MD simulations predict that H atom scattering is surface specific due to the reduced influence of the random force.

## Abstract 2

We derive a many-body formalism for interaction energies of adsorbates in metals and use it for. electronically non-adiabatic H atom scattering simulations from metal surfaces.

## Graphical abstract

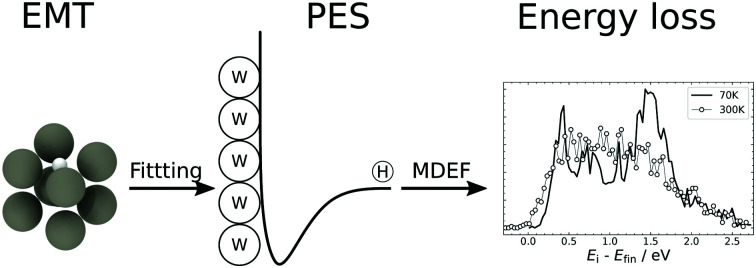

## 1 Introduction

Adsorption is a prerequisite to most surface chemistry and requires that the incident molecule transfers kinetic energy to the solid, either *via* excitations of phonons or electron–hole pairs (ehp). H-atom adsorption on transition metals is of special interest^1–4^ as the efficiency of energy transfer to phonons is reduced, a result of the light mass of hydrogen compared to the surface atoms. This makes an accurate description of ehp excitation essential and H atom scattering from metal surfaces an excellent test case for modeling electronically non-adiabatic dynamics beyond the Born–Oppenheimer approximation.^5^

Two theoretical frameworks to accomplish this have evolved over the last decades: (i) independent electron surface hopping^6^ and (ii) mean-field methods like the effective Hamiltonian approach^7,8^ or molecular dynamics with electronic friction.^9–11^ Electronic friction—the most commonly used approach for H atom interactions with metals—treats the electrons as a bath, which is well-suited to describe a metal's electronic continuum.^10,11^ The coupling between the translational degrees of freedom of the atom and metal electrons is then described by a frictional drag force upon the classically moving nuclei. The friction tensor is commonly treated simply as a coefficient, which can then easily be calculated from the background electron density at the location of the nuclei. This is referred to as the local density friction approximation (LDFA).^10–13^ Using this model of ehp excitations, a Langevin equation is used to propagate classical trajectories. This introduces a temperature dependent random force that ensures detailed balance.^14^ Despite the LDFA works well in modeling the ehp influence on the dynamics of atoms at metal surfaces, it is not applicable for molecule-surface scattering. The contribution due to the molecular electronic structure into the friction can be taken into account by the orbital-dependent friction approach.^15,16^

The critical step in carrying out molecular dynamic simulations with LDFA electronic friction is the simultaneous acquisition of reliable configuration–dependent energies and background electron densities. Many-body potentials like the Embedded Atom Method (EAM)^17,18^ or Effective Medium Theory (EMT)^19–22^ have the advantage that their energy expressions contain an electron density model, allowing potential gradients and LDFA based friction coefficients to be computed on the fly. By parameterizing an EMT expression by fitting to DFT data, full-dimensional potential energy surfaces (PES) and electron density functions can be derived.^23^ In recent studies, this approach was used to investigate the scattering dynamics of H and D atoms from six late fcc (111) transition metal surfaces and comparisons of predicted energy losses were in excellent agreement with experiment.^23–25^ Remarkably, the scattering dynamics of H and D from these six metals were quite similar, prompting the authors to speak of “universal behavior”.^24^

In this work, we investigate how the surface and crystal structure influence the H and D translational energy losses resulting from collisions at metal surfaces. This required us to extend the EMT formalism^22^ to the bcc crystal symmetry, the formalism for which we also present. We parameterized these newly derived energy formulae by fitting them to *ab initio* energies for two bcc metals, Mo and W, both with (111) surface structures. We also showed that the same EMT parameters accurately describe the H interactions with the (110) surfaces of W and Mo. Finally, we used the PESs and electron densities to perform LDFA electronic friction molecular dynamics simulations of H atoms scattering and computed energy loss distributions. We find, as before, that there are only small differences in the H atom energy losses when comparing different metals. However, scattering from different facets—even for the same metal—leads to significantly different scattering dynamics. In contrast to (111) facets, H scattering from (110) facets leads to deep H atom penetration followed by scattering back to the vacuum. This produces a large energy loss that should be observable in experiment. We make the prediction that H scattering from W(110) at liquid nitrogen temperature is the best possibility to observe this novel scattering process.

## 2 Theory

### 2.1 Effective medium theory

The Effective Medium Theory (EMT) has proven useful to describe gas-surface interactions for fcc metals.^2,14,22–24,26–30^ Here, we extend the previously formulated EMT formalism to the case of bcc metals.

EMT represents the energy of a real system relative to a reference system.^22^ Hence, the total energy *E* of a system consisting of *N* atoms is the sum of the energy of the reference system and a correction term Δ*E*:1
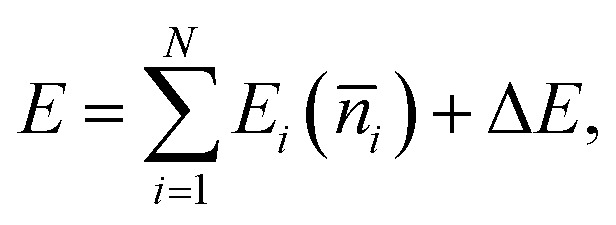
where *E*_*i*_ (*n̄*_*i*_) represents the cohesive energy of atom *i* and depends on the average background electron density *n̄*_*i*_ surrounding the atom. *E*_*i*_ (*n̄*_*i*_) is calculated by considering atom *i* to be an impurity embedded in a metal host. Jacobsen *et al*.^22^ and Janke *et al*.^2^ chose a perfect fcc crystal as a reference system, but other choices are possible. In our new formalism, we choose a perfect bcc crystal to serve as an effective medium, and follow the derivation used for fcc metals.^22^

The correction term Δ*E* is often represented in the following form:2
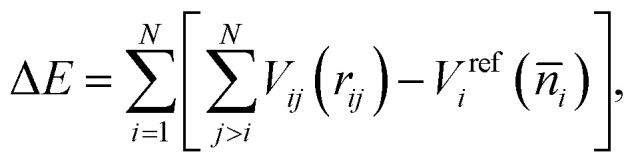
where *V*_*ij*_(*r*_*ij*_) is the pairwise correction term due to the interaction between atoms *i* and *j* separated by the distance *r*_*ij*_. *V*^ref^_*i*_ (*n̄*_*i*_) is the many-body correction term for the reference system. The background electron density *n̄*_*i*_, averaged over the volume inside a sphere with the radius *s*_*i*_, serves as a connection between the real system and the reference system and is calculated as3
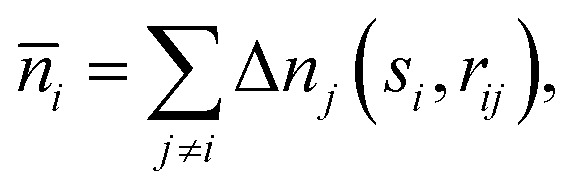
where Δ*n*_*j*_(*s*_*i*_,*r*_*ij*_) is the electron density tail of atom *j* contributing to the background electron density in the location of atom *i*. These density tails can be approximated by exponential functions resulting in the following equation:4
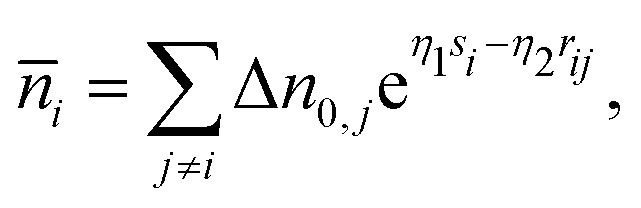
where *η*_1_ and *η*_2_ describe the fall-off of the many-body and the pairwise contributions to the average electron density *n̄*_*i*_, respectively. Δ*n*_0,*j*_ is assumed to be a constant. On the other hand, the DFT calculations on the level of local density approximation lead to the following relation:^21^

5 *n̄*_*i*_ = *n*_0_e^−*η*(*s*_i_−*s*_0_)^,

where *s*_0_ defines a sphere of the same volume as the Wigner–Seitz cell of a perfect fcc or bcc crystal in equilibrium. Setting *s*_*i*_ = *s*_0_ and assuming that only nearest neighbors contribute to the background electron density, eqn (4) and (5) give6
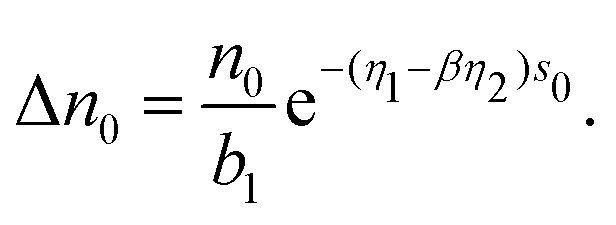
Here, *b*_1_ denotes the number of nearest neighbors. The geometric factor *β* relates the neutral sphere radius *s*_0_ to the nearest-neighbor equilibrium distance

7 *r*_1_ = *βs*_0_.

In general, it can be shown that the interatomic distance to the neighbors situated in the *q*-th shell in a perfect lattice is given by

8 *r*_*q*_ = *d*_*q*_*βs*_0_.

For the fcc metal the coefficients *d*_*q*_ are related to the number of shell *q* by a simple formula9
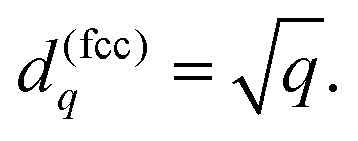
It is slightly more complex for the bcc lattice: the values of *d*^(bcc)^_*q*_ can be calculated numerically with the aid of the primitive lattice vectors. Table 1 shows the radii and the number of atoms for the first 10 shells.

**Table 1:** Radius *d*_*q*_ of the *q*-th shell in the units of *βs*_0_ and the corresponding number of the atoms *b*_*q*_ belonging to it for both the fcc and bcc crystal

*q*	*d* ^fcc^_*q*_	*b* ^fcc^_*q*_	*d* ^bcc^_*q*_	*b* ^bcc^_*q*_
1	1	12	1	8
2	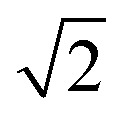	6	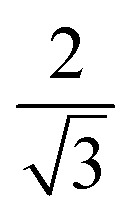	6
3	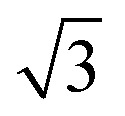	24	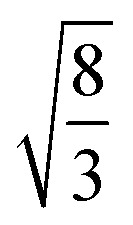	12
4	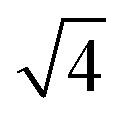	12	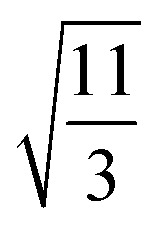	24
5	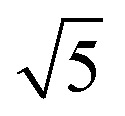	24	2	8
6	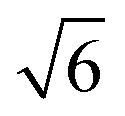	8	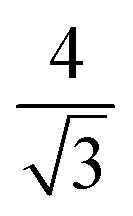	6
7	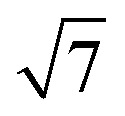	48	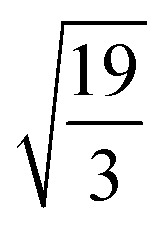	24
8	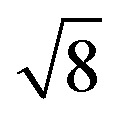	6	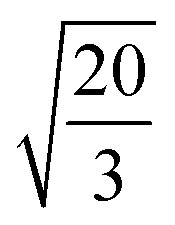	24
9	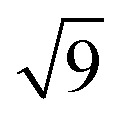	36	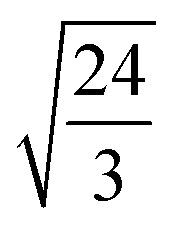	24
1	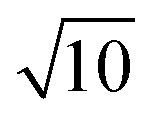	24	3	32

The geometrical factor *β* entering eqn (8) is defined by:10
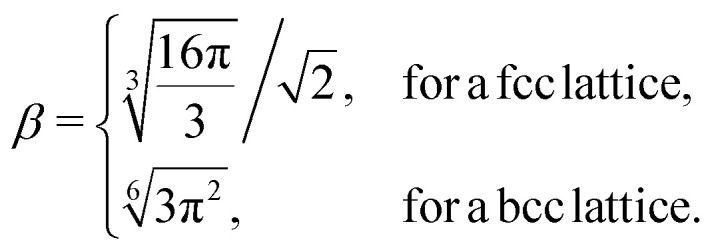


Substituting eqn (6) into eqn (4) and comparing with eqn (5) we obtain the equation:11
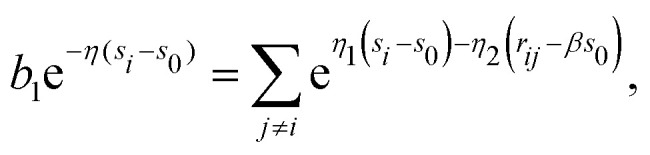
which implies, for the sake of consistency, that

12 *η* = *βη*_2_ − *η*_1_.

Rose *et al*.^31^ developed a functional producing the cohesive energy for a crystal lattice of the following form

13 *E*_*i*_ = *E*_0_ [1 + *λ* (*s*_*i*_ − *s*_0_)]e^−*λ*(*s*_*i*_−*s*_0_)^ − *E*_0_.

The expression for the neutral sphere radius *s*_*i*_ can be obtained from eqn (11):14
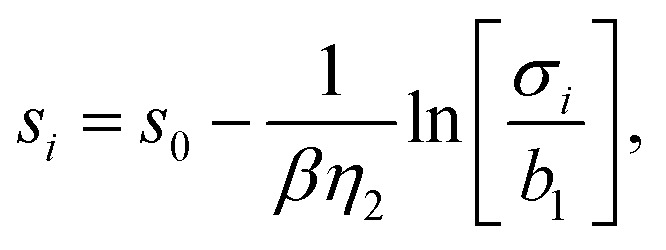
with *σ*_*i*_ being the short hand notation for15
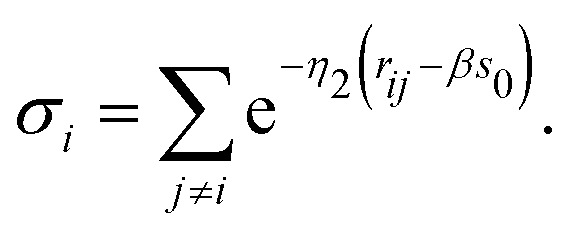
*E*_0_ is the cohesive energy for the equilibrium geometry. The pairwise correction term and the potential energy of the reference system entering eqn (2) can be represented in the following form16
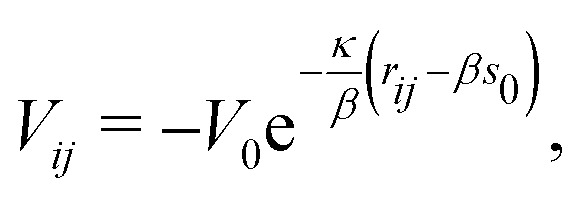
and

17 *V*_ref_ = −*b*_1_*V*_0_e^−*κ*(*s*_i_−*s*_0_)^,

respectively.^22^

The above formalism allows the straightforward extension to two-component systems like metal alloys or a hydrogen atom at metal surfaces. Then, the total cohesive energy of the system consists of the sum of the partial (species-specific) cohesive energies18
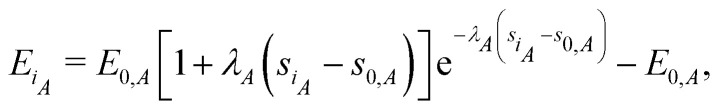
where index *A* labels species *A*. In a two-component system the neutral sphere radius of atom *i*_*A*_ belonging to species *A* is defined by the following formula:19
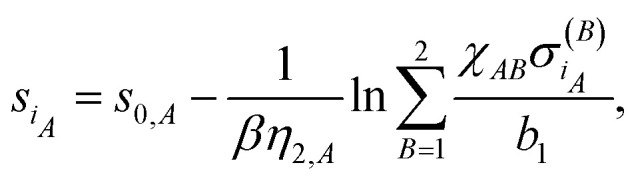
where index *B* runs over the species. The important difference between eqn (19) and (14) resides in the quantity20
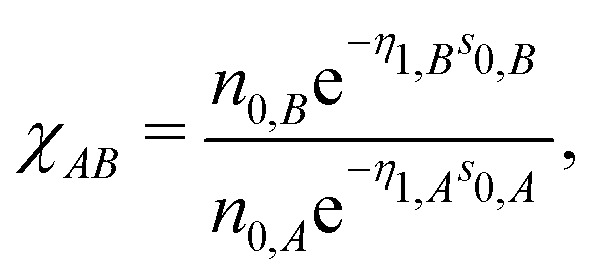
which accounts for the contribution of cross-terms between two different species to the neutral sphere radius. Note, that *χ*_*AA*_ = 1 in the case of *A* = *B*.21
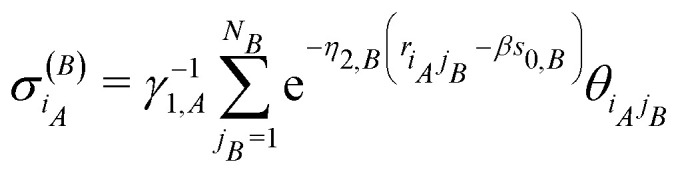
is the sum of exponential pair-wise contributions of the atoms belonging to species *B* to the neutral sphere radius *s*_*iA*_. The sum in eqn (21) runs over all atoms of species *B*, and in case of *B* = *A* the self-interacting term (*j*_*A*_ = *i*_*A*_) is excluded from the sum.

The pairwise correction term in eqn (2) is constructed in a similar way to eqn (21),22
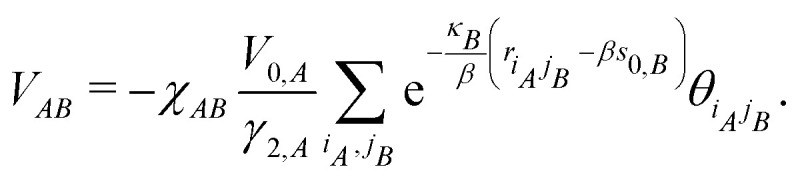
Finally, the reference energy contribution to eqn (2)23
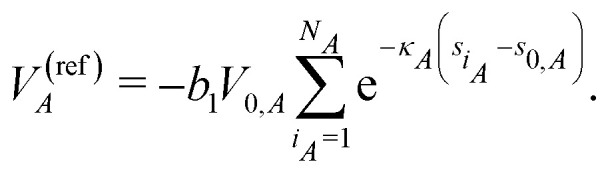
is defined as in eqn (17).

The factor24
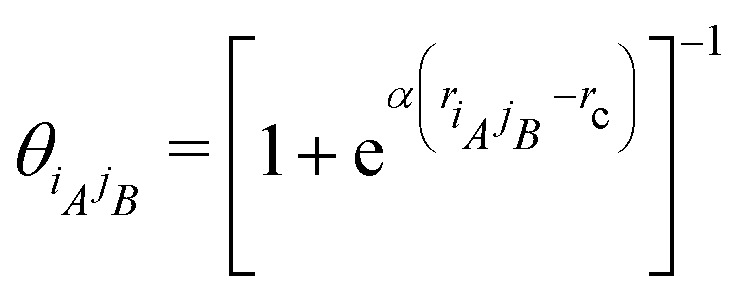
in the formulas above serves as a smooth cut-off function needed for molecular dynamics simulations.^2^ The falloff parameter25
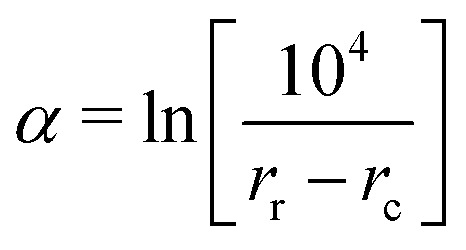
dictates the steepness of the cut-off function, *r*_c_ = *r*_3_ is the cut-off radius set to the third-nearest neighbor distance in equilibrium, and26
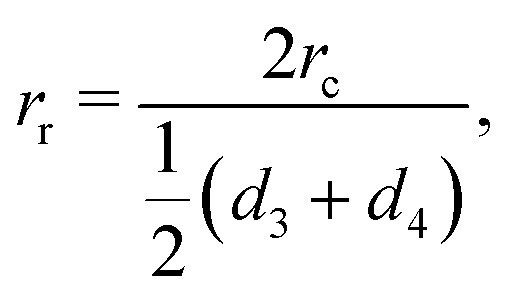
where *d*_3_ and *d*_4_ are given in Table 1. The normalization coefficients27
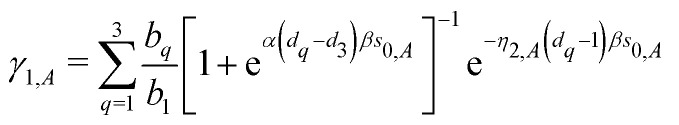
and28
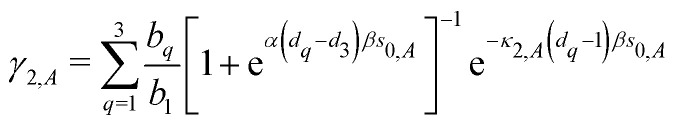
in eqn (21) and (22) ensure that for the perfect bulk structure the total energy is zero.^2,22^ The sums in the above equations run over the first three shells. *b*_*q*_ is the number of atoms in shell *q*. For a perfect fcc crystal *b*_1_ = 12, *b*_2_ = 6, and *b*_3_ = 24, while for a bcc crystal *b*_1_ = 8, *b*_2_ = 6 and *b*_3_ = 12.

EMT characterizes each atomic species in the system with seven parameters: *E*_0_, *n*_0_, *s*_0_, *λ*, *η*_2_, *V*_0_ and *κ*. All parameters except for *n*_0_ are connected to bulk properties that can be obtained experimentally.^2,21,22^*E*_0_ is the cohesive energy. *s*_0_ is related to the lattice constant *a*_0_ by expressions:29
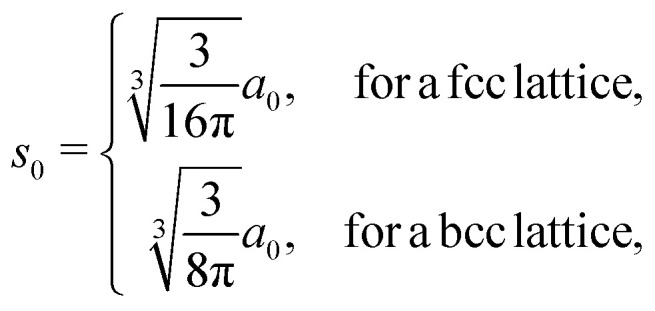
which were obtained from eqn (8) noting that *a*_0_ = *r*_2_ for both fcc and bcc lattice.

The remaining parameters *λ*, *V*_0_, *η*_2_ and *κ* are related to the elastic moduli of a metal (see Appendix).

### 2.2 Electronic structure calculations

The optimal EMT parameters must be found by fitting the EMT energy function to energy values obtained from *ab initio* calculations, using a large number of configurations. These were determined using VASP5.3.5^32–35^ with the PBE functional^36,37^ and with the electron-core interactions treated within the framework of the projector-augmented wave (PAW) approach.^38^ The plane-wave basis set cutoff energy was set to 400 eV. Partial occupancies were modeled with the method of Methfessel–Paxton^39^ (*N* = 1) with a smearing width of 0.1 eV. We calculated the energy for both (111) and (110) facets of W and Mo metals. The simulation cell contained a (2 × 2) six-layered slab with the bottom layer held stationary. The *k*-point grid for the Brillouin zone for W(111) and Mo(111) was sampled with the (6 × 6 × 1) Monkhorst–Pack mesh,^40^ while for W(110) and Mo(110) the (12 × 12 × 1) and the (10 × 10 × 1) mesh were used, respectively.

**Fig. 1:**
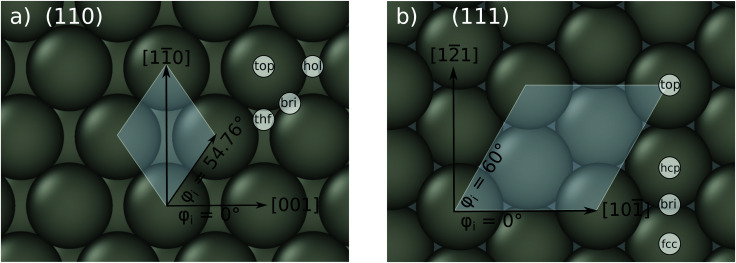
Top view on the bcc (110) surface, shown in panel (a), and the bcc (111) surface in panel (b). The cartesian coordinate system, the incidence azimuth and the most important high symmetry sites are also shown. The white shaded areas mark the *p*(1 × 1) unit cell.

The system geometries were sampled in two ways: (i) H atoms were placed at nodes of a 3D grid consisting of about 1000 points, while the metal atoms were fixed at their equilibrium lattice positions (Fig. 1); (ii) configurations were taken from *ab initio* molecular dynamics trajectories simulating the scattering of an H atom from a surface at 120 K. The initial positions of H atom for these AIMD simulations were set to be 6 Å above the surface at random lateral coordinates. The initial velocity of the H atom was set to correspond to the incidence kinetic energy of 5 eV and the incidence angle of 30°. The time step was set to 0.1 fs and the H atom was considered to be scattered when it was more than 6.05 Å above the surface. The initial positions and velocities of the surface atoms were sampled from the equilibrium *NVE* MD simulations of a metal slab at 120 K. The snapshots were taken from a 1 ps MD trajectory with an interval of 100 fs.

### 2.3 Fitting procedure

A genetic algorithm developed in our group^23^ was used to fit the EMT function to the DFT energies described above. We used the relations of the fitting parameters to the bulk properties of metals discussed in Subsection 2.1 to constrain values of the following parameters. *s*_0,M_ was calculated from eqn (29) using the lattice constant *a*_0_ obtained from the DFT calculations. The cohesive energy of the metal *E*_0,M_ was set to its experimental value.^41^*λ*_M_ was set to a value ensuring a good agreement with the literature value of the bulk modulus.^41^ This leaves eleven parameters remaining, which were optimized to fit the DFT data. We also used MD simulations to check that the metal slab remained intact up to 900 K for 100 ps. Finally, we compared the EMT background electron density to that of the DFT calculations. Although these two physical quantities are not strictly comparable, they agree well within one another.

### 2.4 Non-adiabatic molecular dynamics simulations

We treat electronically non-adiabatic effects in terms of a drag force and a random force, using the Langevin equation to govern the motion of the H atom30
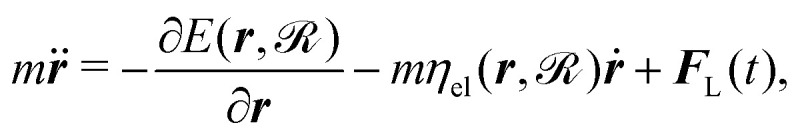
Here, *m* and ***r*** are the projectile's mass and position, respectively; 
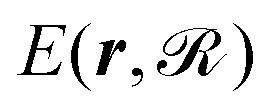
 is the ground-state potential energy surface provided by the optimized EMT energy expression that depends not only on the projectile coordinates ***r*** but on the coordinates of the surface atoms 
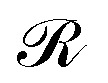
. Eqn (30) can be derived from the time-dependent Schrödinger equation using a mean-field approximation in the limit of weak non-adiabatic couplings.^42^ In that case, the friction term 
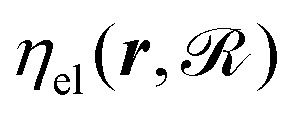
 is obtained by Fermi's golden rule with perturbations defined first-derivative non-adiabatic couplings of the electronic states. In this work, we are operating within the framework of the local density friction approximation (LDFA),^10–12^*i.e.*, we are dealing with a single friction coefficient instead of a friction tensor. The friction coefficient is calculated with the aid of the background densities associated with the EMT-PES. The detailed mapping procedure between friction coefficient and background electron density is described elsewhere.^2^ The random force ***F***_L_ (*t*) is modeled by a stochastic process with a Gaussian white noise of zero mean value

31 〈***F***_L_ (*t*)〉 = 0,

and the variance being characterized by the second fluctuation-dissipation theorem^43^32
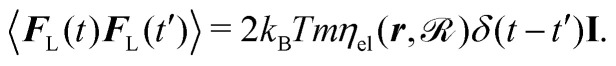
Here, **I** denotes the 3D unity matrix and *T* is the surface temperature. We emphasize that neglecting the random force, as has sometimes been done,^44^ can lead to spurious results.^14^ The EMT-PES and the Langevin propagator integrating eqn (30) are implemented in our homemade program *md_tian2* available at a public repository.^45^

The MD trajectories simulating H scattering from a metal surface were started with a H atom placed at 6 Å above the surface with a lateral position chosen randomly. The time step was 0.1 fs and the trajectory was stopped once the projectile was more than 6.05 Å above the surface. The metal surface was equilibrated to 70 K and 300 K in the following way: for 100 ps the slab was equilibrated with an Anderson-thermostat,^46^ and then propagated microcanonically for additional 100 ps, using the velocity-Verlet algorithm.^47,48^ Afterwards, we ran a 1 ns-equilibrium trajectory and took a snapshot every picosecond. These shapshots sample the equilibrium slab geometries at the desired temperature which served as slab initial conditions for the scattering dynamics simulations.

## 3 Results and discussion

### 3.1 Full dimensional PES for H on W(111) and Mo(111)

**Fig. 2:**
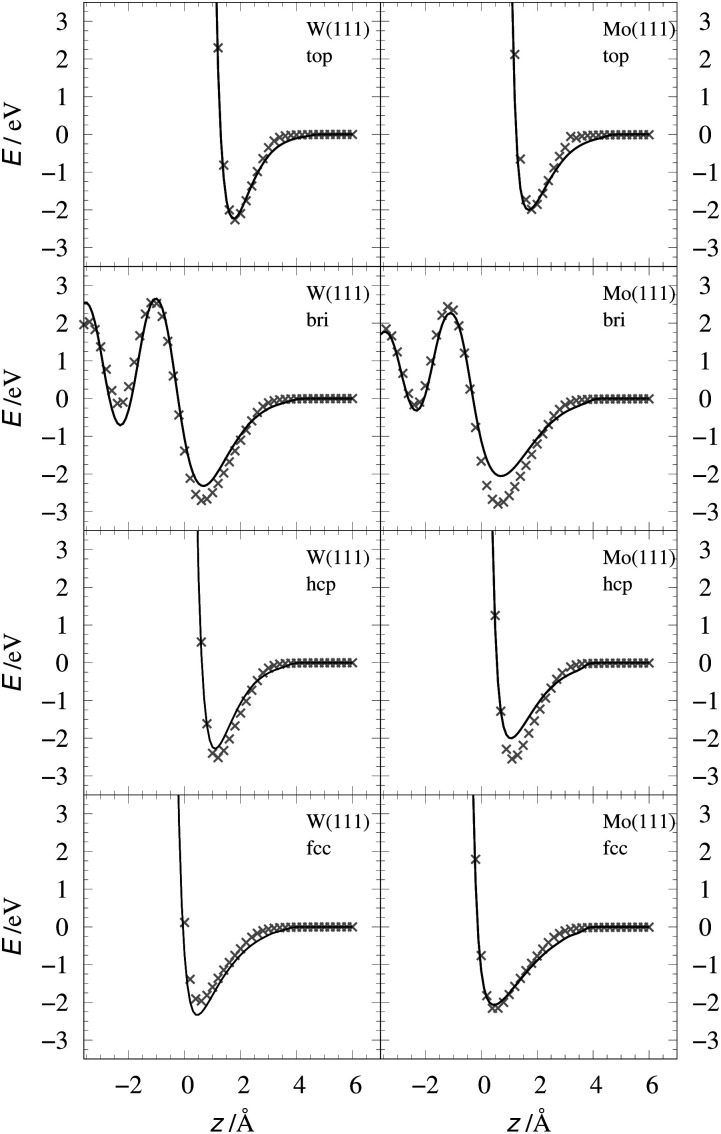
Interaction energy of H atom with the metal as a function of the projectile's height *z* over the surface shown for several high-symmetry sites (see Fig. 1). The gray crosses mark the DFT energies of H/W(111) and H/Mo(111), which served as input data for the fit. The black line represents the EMT fitting function. Note that the metals were held fixed at their lattice coordinates.

**Fig. 3:**
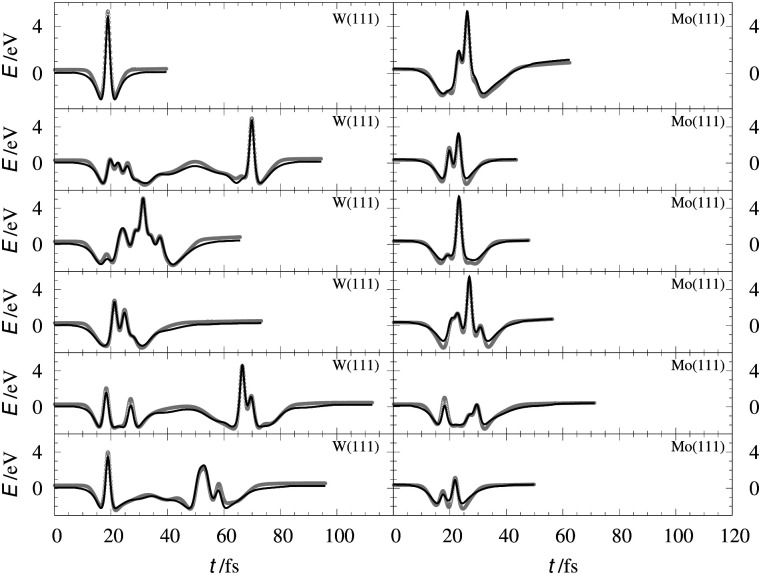
Interaction energies corresponding to configurations sampled from AIMD trajectories. The gray crosses represent the *ab initio* energies along the trajectories for H/W(111) (left panels) and H/Mo(111) (right panels), respectively. The black line stands for the energy from EMT fitting function.

**Fig. 4:**
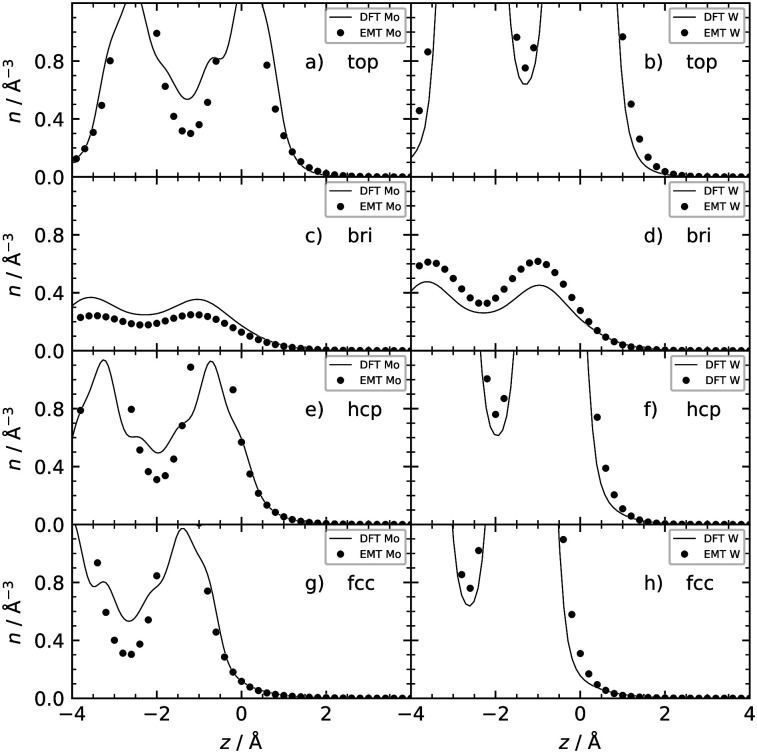
Background EMT and DFT electron densities for Mo(111) and W(111) as a function of the H atom height over the surface at four different high-symmetry sites shown in Fig. 1a. Note that the metal atoms were kept fixed at their equilibrium lattice coordinates.

Table 2 shows the optimized EMT parameter sets for atomic hydrogen interacting with both W(111) and Mo(111). Fig. 2 shows cuts through the EMT-PESs for the two metals. The root mean-square error (RMSE) for H/W(111) and H/Mo(111) is 0.25 eV and 0.26 eV, respectively. Fig. 3 shows comparisons of EMT-PESs to DFT results as AIMD trajectories that include structures with surface atoms displaced from their equilibrium positions. The resulting EMT-PESs for H/Mo and H/W show an overall RMSE of 0.27 eV and 0.30 eV, respectively. Fig. 4 shows cuts of the EMT background electron density 
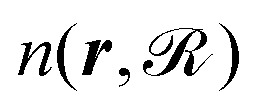
 for four surface symmetry sites along the surface normal, as well as the corresponding electron densities obtained from the DFT calculations absent the H atom. Again, the agreement is good.

**Table 2:** EMT parameters defining the H/W and H/Mo interaction energies and background electron density

	*η*_2_/Å^−1^	*n*_0_/Å^−3^	*E*_0_/eV	*λ*/Å^−1^	*V*_0_/eV	*κ*/Å^−1^	*s*_0_/Å
W	3.546	0.051	−8.90	3.505	1.518	2.296	1.564
H	7.049	0.141	−3.36	7.701	0.482	8.047	0.680
Mo	2.782	0.051	−6.82	3.738	2.595	3.899	1.554
H	5.371	0.066	−2.33	6.236	0.407	8.767	0.844

### 3.2 EMT-PES transferability to the (110)-facet

**Fig. 5:**
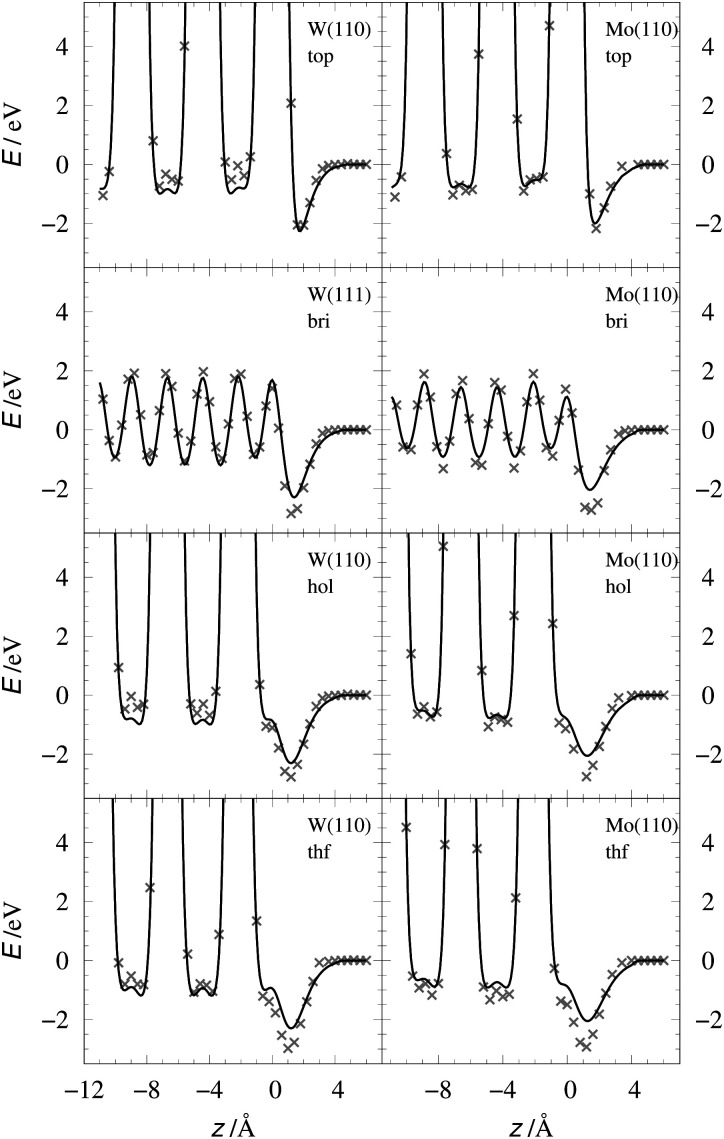
EMT energy dependence on z coordinate of the H-atom at W(110) (left panels) and Mo(110) (right panels) shown for several high-symmetry sites. The gray crosses mark the corresponding DFT energies. Note, the EMT-PES was fitted to the (111) data.

**Fig. 6:**
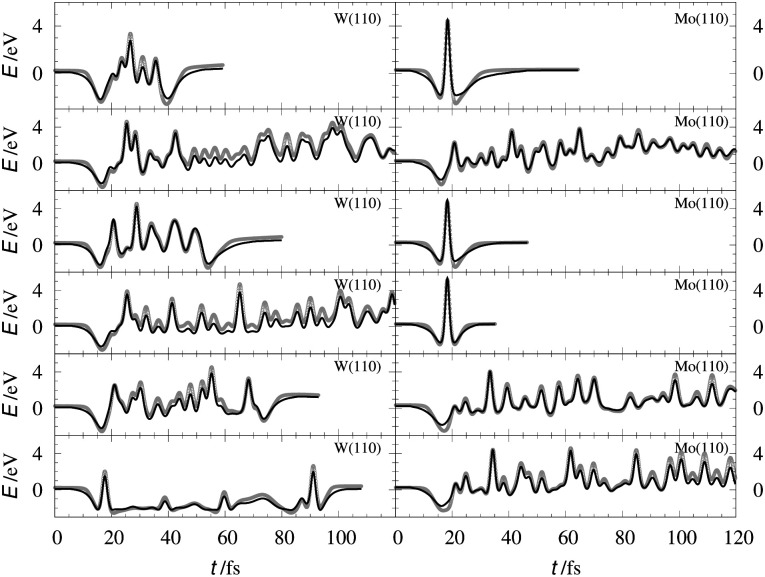
H/W(110) and H/Mo(110) interaction energies calculated for the configurations sampled from AIMD scattering trajectories. The gray crosses represent the DFT energies. The black line stands for the EMT-PES.

The EMT energy expression is independent of the surface facet; hence, the EMT parameters of Table 2 can just as easily be used to produce a PES for H interacting with a (110) surface. This is an advantage over other methods like neural networks, which need to be retrained for each facet. Fig. 5 and 6 show comparisons of DFT data to the EMT energies for H on W and Mo(110) facet. Agreement between the (111)-fitted EMT-PES and DFT is good. We emphasize that these comparisons sample a wide variety of configurations including those corresponding to single bounce scattering as well as penetration of H atom into the bulk. In all cases the EMT-PESs are in a good agreement with the DFT calculations with the RMSE of about 330 meV (13.2 meV per atom) without no adjustment to the fitting parameters.

**Fig. 7:**
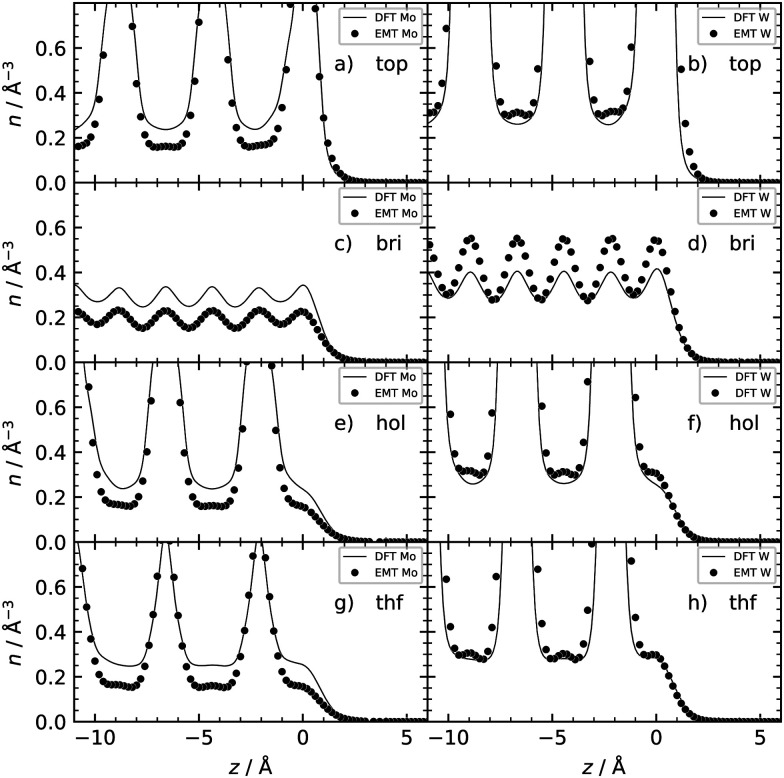
Background EMT and DFT electron densities for Mo(110) and W(110) as a function of the H atom height over the surface at four different high-symmetry sites shown in Fig. 1b.

We also checked the accuracy of the EMT electron densities against DFT calculations—see Fig. 7. As in the (111) case, agreement is good. In case of H/Mo(110), the EMT background electron density (filled circles) is systematically ∼30% lower than the one from DFT (solid line). However, this does not influence the predicted energy loss distributions appreciably.

Nowadays, it is possible to craft Neural-Network potentials with fitting errors (RMSE) less than 1 meV per atom.^49^ So, our potentials may seem by comparison inaccurate. But the high accuracy of Neural-Network potentials comes at a cost of complexity. It requires far more DFT data to be trained, and it must be retrained from facet to facet. Furthermore, it delivers no background electron density information necessary for computing electronic friction forces. The EMT approach presented here is by comparison extremely simple, transferable between facets and, as has been shown, despite the reduced accuracy in reproducing DFT data, accurate enough to reproduce experimental energy loss distributions.^24^ Another strength of the EMT-PES is that the projectile cannot enter out-of sampling regions of the configuration space during MD simulations—an aspect which needs to be always checked when using Neural-Network potentials.

### 3.3 MD simulations of H scattering

Using the EMT-PESs described above, we performed LDFA frictional based molecular dynamics simulations to compute energy-loss distributions for hydrogen atom scattering from tungsten and molybdenum. We launched 10^6^ trajectories with incidence energy of *E*_in_ = 2.76 eV and incidence angle *ϑ*_in_= 45°. To reflect typical experimental conditions,^50^ we selected trajectories scattered at the specular angle with the in-plane and out-of-plane tolerance of ±5°. We refer to these distributions as specular energy loss distributions.

**Fig. 8:**
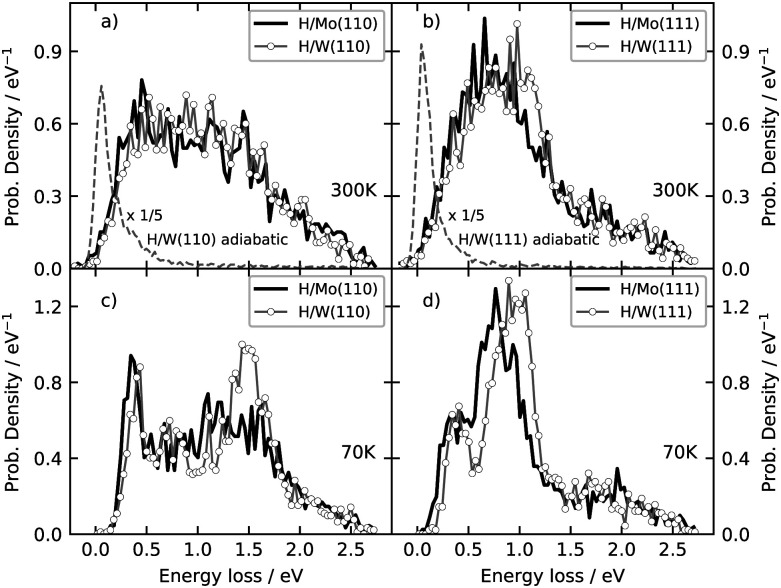
Specular energy loss distributions of H atoms scattered from molybdenum and tungsten surfaces. Upper panels show results for the surface temperature of 300 K and lower panels for 70 K. The gray dashed line represent adiabatic simulations for H atom scattering from tungsten, demonstrating the crucial contribution of electrons to the energy loss of the scattered particles. The initial conditions are*: E*_in_ = 2.76 eV, *ϑ*_in_ = 45°, and *φ*_in_ = 0° (see Fig. 1).

Fig. 8 shows simulations for H scattering from both surface facets of Mo and W at 70 K and 300 K. The energy loss distribution obtained from electronically adiabatic MD simulations are also shown. The MDEF simulations predict a much larger energy loss dominated by ehp excitation. The mean energy losses are all about 1 eV, consistent with experimental observations for H atom scattering from fcc metals^24^ and similar in magnitude to predictions of another calculation using a reduced dimensional PES.^51^ When comparing the four scattering calculations at two temperatures, we see that there is very little difference in the energy loss distributions for Mo and W, when other factors are the same. On the other hand, there is a distinct difference in the energy loss distributions when comparing different surface facets or different temperatures. The effect of temperature has been reported previously^14^ and arises from the reduced influence of the random force at low temperature.

**Fig. 9:**
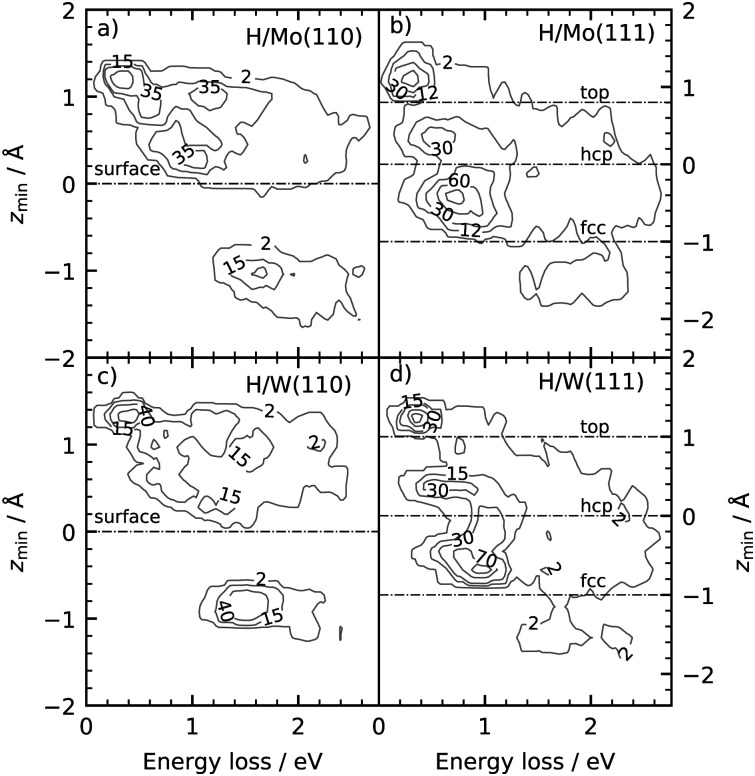
Distribution of specular scattering events as a function of the energy loss and the depth of penetration of H atom scattered from (a) Mo(110), (b) Mo(111), (c) W(110), and (d) W(111). The surface temperature is 70 K. The other conditions are the same as in Fig. 8. The signal above the black, dashed line indicate from which layer the projectiles repelled. The labels top, hcp and fcc refer to the high-symmetry sites of the (111) facet and are shown in Fig. 1(b). The bin sizes are 0.027 eV and 0.063 Å.

The differences seen in the H-atom energy loss distributions for different surface facets—compare Fig. 8(a)–(d)—are due to differences in surface structure. This can be inferred from results presented in Fig. 9. Here, contour plots report the number of specular scattering events as a function of the energy loss and depth of penetration for trajectories of panels Fig. 8(c) and (d), where the surface temperature was 70 K. A clear correlation between energy loss and the depth of penetration is seen for both surface geometries—the deeper the H atom moves into the bulk, the more kinetic energy is lost to the metal.

This can be qualitatively understood from the structures of the surfaces. The (111) surfaces allow access to three surface sites—top, fcc-hollow and hcp-hollow—broadening the energy-loss distribution as the three sites allow for different degrees of surface penetration. For (110) surfaces, the surface density is higher—over 70% of the specular scattered H atoms do not penetrate the surface. But, the (110) facets also exhibit geometric channels that allow very deep penetration that results in a better resolved high energy loss feature in the energy loss distributions. It is noteworthy that subsurface-penetration scattering processes are predicted by these calculations, and hints are provided how these might best be observed experimentally. Specifically, we suggest that H atom scattering experiments using W(110) held at liquid nitrogen temperature would provide clear signatures of subsurface scattering. Tungsten is more favorable to these proposed experiments and it exhibits higher background electron density (see Fig. 4): resulting in higher values of the friction coefficient, which in turn leads to larger energy losses for deep penetration.

### 3.4 MD simulations of H adsorption

Finally, we report the sticking probabilities for H under the incidence conditions of this work—see Table 3. Remarkably, the sticking probability is uniformly about 0.4 regardless of the identity of the metal, the surface facet or the temperature. This reflects the mechanism of adsorption previously identified for H adsorption to Au(111).^2,4^ In this mechanism, adsorption results from trajectories that sample the high electron density below the surface of the metal and subsequently resurface with less than enough energy to desorb. In our case, for the (110) surface, resurfacing originates predominantly from the underlying subsurface and—to a minor extend—from the third layer, while for the (111) surface the resurfacing occurs even from the sixth metal layer. This strong migration reflects the small distance between the individual layers in the (111) surface along with a variety of easy accessible diffusion pathways due to the low packing density.

**Table 3:** Sticking coefficient *S*_0_ computed from the same set of trajectories that were used for the calculation of the specular energy loss distributions shown in Fig. 8

System	300 K	70 K
H/Mo(110)	0.44	0.44
H/Mo(111)	0.40	0.41
H/W(110)	0.42	0.41
H/W(111)	0.40	0.40

## 4 Conclusion

In summary, we have extended the EMT formalism derived for fcc metals^22^ to the bcc case. We then fit the newly derived formulae to DFT data for H interacting with W and Mo, which led to full dimensional PESs and electron densities. We employed the PESs and the electron densities to carry out electronically non-adiabatic MD simulations of H atom scattering, following previous work that used the LDFA approximation with a Langevin propagator. Specifically, we predict energy loss distributions for H scattering from (111) and (110) facets of these two metals at 2.76 eV incidence energy. Although no experiments are currently available for bcc metals, our results are similar to what has been seen for H scattering from fcc metals. This suggests that the current results are likely to be a reliable prediction of experiment. We find only subtle differences in the energy loss distributions arising from the scattering of H atom with these two metals; however, scattering from the (111) and (110) facets are distinctly different. Remarkably, on the (110) facet, we predict a clearly resolvable energy loss peak that arises from sub-surface scattering. The calculations predict that the subsurface scattering is most easily seen for H scattering from W(110) at reduced surface temperatures.

## Conflicts of interest

There are no conflicts to declare.

## Supplementary Material

## Appendices

### Derivation of elastic constants with contributions from the first shell

The components of the elastic tensor can be obtained as follows:

33

where *Ω*_0_ is the volume per metal atom at equilibrium conditions, *ε* is the energy per atom, *r*_*k*,*ρ*_ is the Cartesian component *ρ* of the position vector of neighbor *k*, *N*_B_ is the total number of neighbors. In case of a fcc lattice it is sufficient to include only the neighbors located in the first shell. For a bcc lattice on the other hand, it is necessary to include the second shell, too. Due to the symmetry of the elastic tensor, defined by eqn (33), there are only three independent components: *C*_11_ = {*C*_*xxxx*_,*C*_*yyyy*_,*C*_*zzzz*_}, *C*_12_ = {*C*_*xxyy*_,*C*_*xxzz*_,*C*_*yyzz*_}, and *C*_44_ = {*C*_*xyxy*_,*C*_*xzxz*_,*C*_*yzyz*_}. The bulk modulus of the system can be obtained with the following relationship that holds for all cubic metals

34

Now, when we insert the EMT energy expression (1) for a one-component fcc metal system into eqn (33), we derive the bulk modulus

35

and the shear modulus

36

Considering only the nearest neighbors, *i.e.* neglecting many-body contributions, one can derive the other elastic constants for a fcc lattice

37

38

and a bcc lattice

39

40

Thus, the bulk modulus reads the same as for the fcc case

41

whereas the third elastic constant for a bcc lattice in EMT is

42

### Accounting the second shell contributions into elastic constants

If a perfect bcc crystal is used as effective medium and only the nearest neighbors are considered, the elastic constants, given in eqn (39), (40) and (42), violate the elastic stability criteria.^52^ As a consequence, we also took the next-nearest neighbors into account. The three elastic constants and the bulk modulus then have the following expressions:

43

44 *C*_12_ = *A* + *Ω* [−*γ*_1_ (*κs*_0_ + 1) + *γ*_2_ (*βη*_2_*s*_0_ + 1)],

45

and

46 *B* = A + *Ω* (−*γ*_1_*κs*_0_ (1 + *M*_*κ*_) + *γ*_2_*βη*_2_*s*_0_ (1 + *M*_*η*_2__)).

The factors *A* and *Ω* are the abbreviation for the following expressions:

47

48

with *M*_*η*_2__ and *M*_*κ*_ being

49

and

50

respectively.
